# Corrigendum to: Integrating Omics and Alternative Splicing Reveals Insights into Grape Response to High Temperature

**DOI:** 10.1093/plphys/kiac047

**Published:** 2022-02-25

**Authors:** Jianfu Jiang, Xinna Liu, Chonghuai Liu, Guotian Liu, Shaohua Li, Lijun Wang

*Plant Physiology*, Volume 173, Issue 2, February 2017, Pages 1502–1518, https://doi.org/10.1104/pp.16.01305

At the time of publication, the authors mistakenly omitted an additional affiliation for Xinna Liu: University of Chinese Academy of Sciences (X.L.), Beijing 100049, China.

